# Fabrication of All-GaN Integrated MIS-HEMTs with High Threshold Voltage Stability Using Supercritical Technology

**DOI:** 10.3390/mi12050572

**Published:** 2021-05-18

**Authors:** Meihua Liu, Yang Yang, Changkuan Chang, Lei Li, Yufeng Jin

**Affiliations:** School of Electronic and Computer Engineering, Peking University Shenzhen Graduate School, University Town, Xili, Nanshan, Shenzhen 518055, China; liumh@pku.edu.cn (M.L.); yang1994@pku.edu.cn (Y.Y.); kcchang@pkusz.edu.cn (C.C.); lilei@pkusz.edu.cn (L.L.)

**Keywords:** GaN, MIS-HEMTs, fabrication, threshold voltage stability, supercritical technology

## Abstract

In this paper, a novel method to achieve all-GaN integrated MIS-HEMTs in a Si-CMOS platform by self-terminated and self-alignment process is reported. Furthermore, a process of repairing interface defects by supercritical technology is proposed to suppress the threshold voltage shift of all GaN integrated MIS-HEMTs. The threshold voltage characteristics of all-GaN integrated MIS-HEMTs are simulated and analyzed. We found that supercritical NH${}_{3}$ fluid has the characteristics of both liquid NH${}_{3}$ and gaseous NH${}_{3}$ simultaneously, i.e., high penetration and high solubility, which penetrate the packaging of MIS-HEMTs. In addition, NH${}_{2}^{-}$ produced via the auto coupling ionization of NH${}_{3}$ has strong nucleophilic ability, and is able to fill nitrogen vacancies near the GaN surface created by high temperature process. The fabricated device delivers a threshold voltage of 2.67 V. After supercritical fluid treatment, the threshold voltage shift is reduced from 0.67 V to 0.13 V. Our demonstration of the supercritical technology to repair defects of wide-bandgap family of semiconductors may bring about great changes in the field of device fabrication.

## 1. Introduction

GaN-based high electron mobility transistors (HEMTs) are good candidates for high frequency and high efficiency power switching applications owing to their attractive superiorities of high breakdown electric field and high saturation electron velocity [1]. Normally-off property is strongly required for GaN devices used in the power electronics systems. To date, there are totally four possible ways to realize enhance-mode (E-mode) GaN devices, which are (a) cascode configuration [2], (b) P-GaN gate GaN HEMT, (c) recessed gate GaN MIS-HEMT or MIS-FET and (d) fluoride implanted gate GaN (MIS-) HEMT. Among them, cascode structure is compatible with Si CMOS platform reducing the production cost and complexity. Furthermore, the Miller capacitance is eliminated because of blocking the reverse recovery diode by GaN devices, thus improving the switching speed and reducing the switching loss [3].

However, a few issues have been reported that may negate the speed advantage in the GaN plus Si hybrid cascode devices, such as increased parasitic inductance [4] and mismatch in intrinsic capacitances between the Si and GaN devices [5]. All-GaN integrated cascode device by replacing the Si MOSFET with a low voltage GaN E-mode device achieved using fluoride ion implantation has proven to be able to address the issues mentioned above and improve the switching speed [3]. However, fluoride ion implantation tends to result in V${}_{th}$ instability and drain current degradation in HEMTs [6]. In addition, fluoride ion implantation requires high energy, and it is difficult to realize general silicon process lines.

Furthermore, metal-insulator-semiconductor (MIS) gate structure is typically adopted to maintain a relatively large gate swing a low gate leakage current. However, there are several raliability issues realted to GaN MIS-HEMTs. When a positive gate bias is applied, defects located in the gate stack act as charge trapping sites. This induces a shift of the device transfer characteristics toward more positive values [7,8].

Generally, the trapping effect in an MIS gate stack could be related to the traps at/near the interface, named interface states/border traps, or in the bulk of the insulator [9]. There have been several techniques used to suppress threshold voltage shift to date, including pre-fluorination argon treatment [10], sputter-deposited Al${}_{2}$O${}_{3}$ [11], in-situ pre-deposition plasma nitridation [12], metal-organic chemical vapor deposition-grown in situ SiN [13,14], hybrid ferroelectric charge trap gate stack [15], etc., to reduce the trapping effect at/near the insulator/semiconductor interface. Due to the low deposition temperature, there also exists large density of traps in the bulk of the gate insulators deposited by PECVD or ALD. Recently, high temperature deposited gate insulator, such as low-pressure chemical vapor deposition (LPCVD) grown SiN${}_{x}$ has been proven to be a robust gate dielectric for both normally-on GaN MIS-HEMTs and normally-off gate recessed hybrid MIS-HEMTs with low bulk trap density. However, the interface quality between LPCVD SiN${}_{x}$ and (Al)GaN is degraded due to the high growth temperature and H erosion. Despite that low temperature deposited insertion layer or N surface plasma treatment have been adopted to improve the interface quality, the drift of V${}_{th}$ still exists in those devices. Supercritical fluid technology can effectively bring elements into materials through supercritical CO${}_{2}$ fluid to reduce trap density because of its penetration and damage-free diffusion ability in the devices [16]. Supercritical technology has been applied in the field of memory [17] and LED [18], but there is no research on the effectiveness of GaN power devices.

In this work, a new way is presented to achieve all-GaN integrated MIS-HEMTs in a Si CMOS platform by replacing the Si MOSFET with a low voltage GaN recessed gate MIS-HEMT. In the process, self-terminated gate open method and quasi-self-alignment technology are adopted allowing the recessed gate was defined and fabricated at the beginning of the process. In addition, we propose the application of supercritical nitridation treatment (SNT) to passivate the defects and mitigate the shift of V${}_{th}$ in the all-GaN MIS-HEMTs. After SNT, the interface trap density in LPCVD Si${}_{3}$N${}_{4}$/AlGaN layer interface is effectively reduced and near 0.13 V shift of V${}_{th}$ in the transfer curve of a GaN power device is observed with a bidirectional gate bias sweep up to 15 V.

## 2. Device Fabrications

The AlGaN/GaN heterostructure was grown by the metal organic chemical vapor deposition(MOCVD) on a Si(111) substrate, which consists of a 4-µm C-doped GaN buffer layer, a 300-nm unintentionally doped GaN channel layer, a 1-nm AlN insertion layer, a 25-nm Al${}_{0.25}$Ga${}_{0.75}$N barrier layer and a 3-nm GaN cap layer for improving surface morphology. On wafer Hall measurement yields a sheet resistance of 363 Ω/square, a 2DEG density of 1.1 × 10${}^{13}$ cm${}^{-2}$, and an electron mobility of 1547 cm${}^{2}$/V·s. The reported devices were fabricated in Founder Microelectronics International Corporation, Ltd, a 6-inch Si CMOS platform.

**Figure 1:**
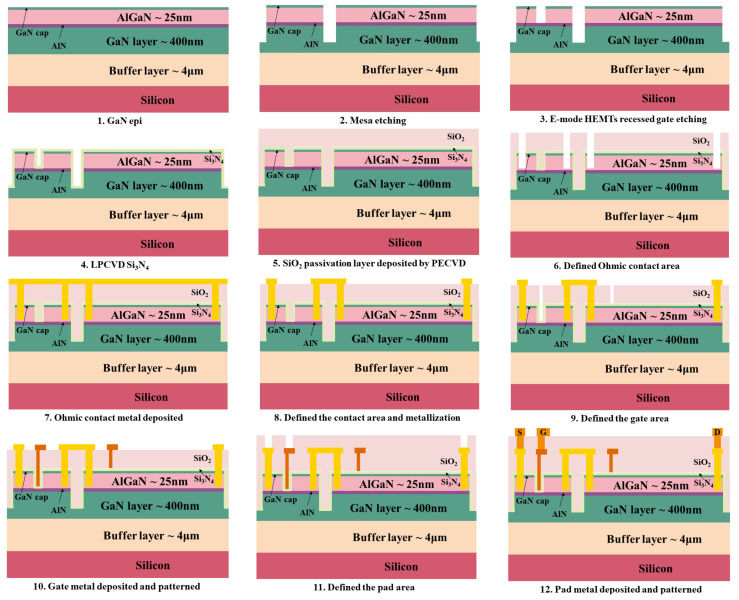
(**1**–**12**) Main process steps of the all GaN integrated MIS-HEMT in CMOS fab.

The main process flow is:

(1) Defining (Figure 1(2)) of the mesa isolation by Cl${}_{2}$/BCl${}_{3}$ based plasma etching.
(2) Patterning (Figure 1(3)) of the recessed gate by etching the AlGaN layer completely.
(3) Deposition of a 35-nm Si${}_{3}$N${}_{4}$ layer using low pressure chemical vapor deposition (LPCVD) (Figure 1(4)). The Si${}_{3}$N${}_{4}$ layer acts as a surface passivation layer and a gate insulator. The LPCVD Si${}_{3}$N${}_{4}$ exhibits good insulating property and passivation effects.
(4) Deposition of a 500-nm oxide layer over the Si${}_{3}$N${}_{4}$ layer by plasma enhanced chemical vapor deposition (PECVD) (Figure 1(5)). The oxide layer acts as the plasma etching sacrificial layer in the follow process patterning source and drain contacts and gate strips, and the gate field plate dielectrics.
(5) Opening (Figure 1(6)) of the source and drain contact windows by etching the oxide layer, the Si${}_{3}$N${}_{4}$ layer and partial AlGaN layer.
(6) Deposition of Ti/Al/Ti/TiN multi metal layers by physical vapor deposition (PVD) as ohmic metal and Patterning (Figure 1(7)) of the source and drain electrode.
(7) Metallization by rapid thermal annealing at 850 ${}^{\circ }$C for 30 s in ambient N${}_{2}$ (Figure 1(8)).
(8) Patterning (Figure 1(9)) of the D-mode gate. In this step, the low power SF${}_{6}$-based inductively coupled plasma (ICP) etching and the buffered HF (BHF) wet etching were adopted sequentially to define the gate stem, realizing a self-terminated dielectric etching (PECVD SiO${}_{2}$/LPCVD Si${}_{3}$N${}_{4}$ etching selectivity is 200:1) on the surface of the LPCVD Si${}_{3}$N${}_{4}$ gate dielectric layer. The self-terminated nature guaranteed good performance uniformity along the whole wafer. Meanwhile, quasi-self-alignment is realized, the E-mode GaN HEMT recessed gate can be fabricated at the same time.
(9) Deposition of TiN/Ti/Al multi metal layers by PVD as gate metal and patterning (Figure 1(10)) of the gate electrode.
(10) After the PAD metal and Final passivation (Figure 1(11),(12)), the devices were annealed at 450 ${}^{\circ }$C for 30 min in ambient H${}_{2}$.

**Figure 2:**
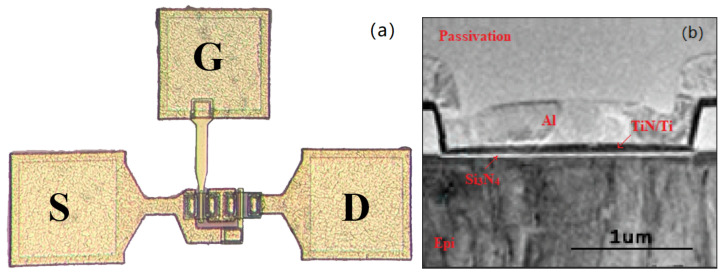
(**a**) Schematic view and (**b**) Cross-section TEM image of the fabricated all-GaN integrated MIS-HEMT.

Figure 1 shows schematics flow of the all-GaN integrated devices The characterization was performed on the devices with a dimension of L${}_{G}$/L${}_{GS}$/L${}_{GD}$/W= 2/4.5/3/24 µm for recessed gate MIS-FET and a dimension of L${}_{G}$/L${}_{GS}$/L${}_{GD}$/W = 1/4.5/8/24 µm for D-mode MIS-HEMT. Figure 2 shows the schematic view and cross-section TEM image of the fabricated all-GaN integrated MIS-HEMT.

## 3. Results and Discussion

All our electrical transport measurements were carried out in an Agilent B1500 semiconductor parameter analyzer and an automated Keithley SCS 4200 system.

### 3.1. Transport Measurements

**Figure 3:**
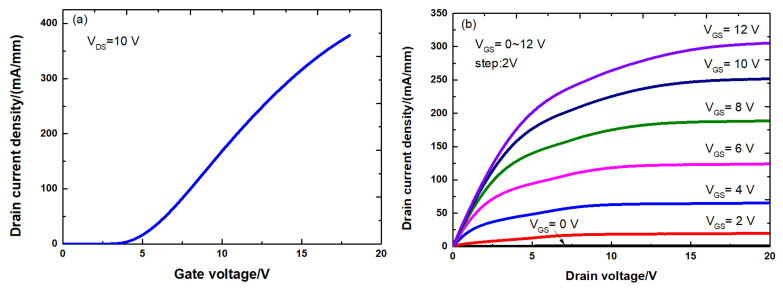
(**a**) DC transfer characteristics and (**b**) output characteristics of the fabricated all-GaN integrated MIS-HEMTs.

**Figure 4:**
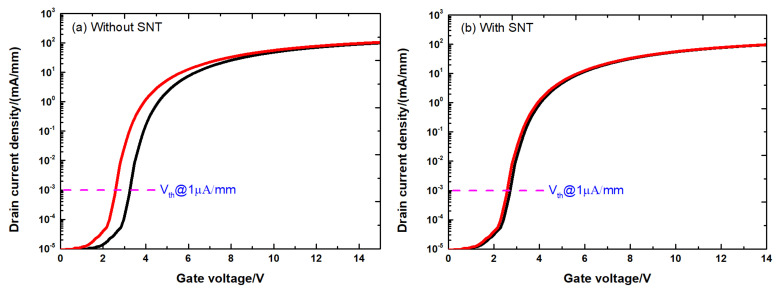
Transfer curves of the all-GaN integrated MIS-HEMTs in didiretional V${}_{GS_{s}weep}$ from 0 V to 15 V and then back to 0 V (**a**) without SNT and (**b**) with SNT.

Figure 3 shows the DC transfer characteristics and output characteristics of the fabricated all-GaN MIS-HEMTs. The V${}_{th}$ of the fresh device is about 2.67 V and the output saturation current is 305 mA/mm at a gate bias of 12 V. The I${}_{DS}$-V${}_{GS}$ transfer characteristics of the all-GaN MIS-HEMTs with and without SNT are shown in Figure 4. All the curves are swept in bidirectional mode at V${}_{DS}$=1 V. For the all-GaN MIS-HEMTs without SNT, significant hysteresis was observed during the sweep, suggesting severe trap-induced V${}_{th}$ shift.

### 3.2. Threshold Voltage Stability

**Figure 5:**
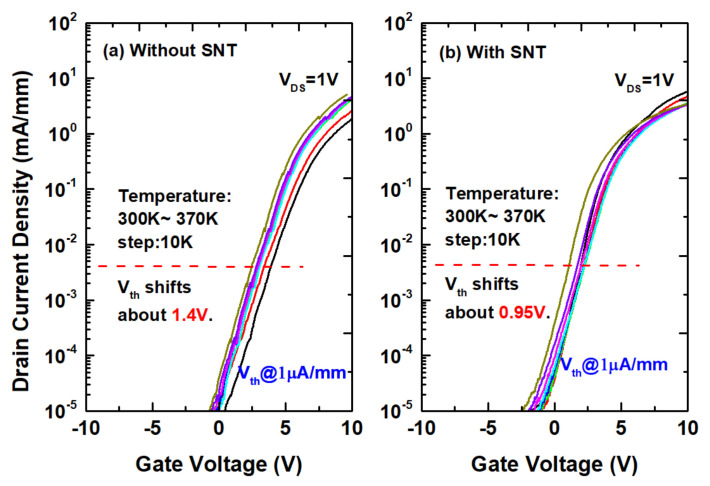
Temperature−dependent transfer characteristics of the all-GaN integrated MIS-HEMTs (**a**) without SNT and (**b**) with SNT.

The temperature dependent V${}_{th}$ hysteresis is evaluated by submitting the all-GaN integrated MIS-HEMTs to thermal simulation from 300 K to 370 K with a 10 K step at V${}_{DS}$ =1 V. Figure 5 shows the transfer characteristics at various temperatures. When the temperature rises from 300 K to 370 K, the threshold voltage shifts about 1.4 V without SNT. Meanwhile, when the temperature rises from 300 K to 370 K, the threshold voltage shifts about 0.95 V with SNT. Compared with that without SNT, the threshold voltage shifts decrease by 0.45 V.

**Figure 6:**
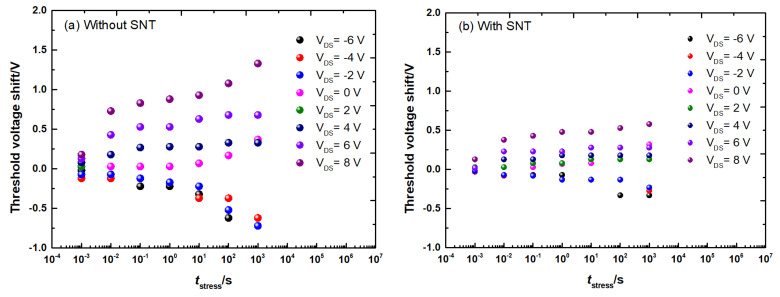
Stress—sequence of the all-GaN integrated MIS-HEMTs (**a**) without SNT and (**b**) with SNT.

To further study the effect of SNT on the shift of V${}_{th}$ after various time intervals and different gate-bias-induced stresses, time-of-fly gate-bias-induced stress and V${}_{th}$ measurement have been performed on the device with both positive and negative gate biases. The shift of V${}_{th}$ with bias-induced stress time from 1 ms to 1000 s at different voltages is shown in Figure 6. The shift of V${}_{th}$ shows a nearly linear relationship with logarithmic time scale, suggesting broad distribution of the time constant of deep traps in the devices without SNT. On the other hand, the shift of V${}_{th}$ in the all-GaN MIS-HEMTs with SNT after the stress is much smaller. The maximum shift of V${}_{th}$ at 8 V gate stress for 1000 s is only 0.58 V.

### 3.3. Si${}_{3}$N${}_{4}$/AlGaN Interface Trap Characterization

**Figure 7:**
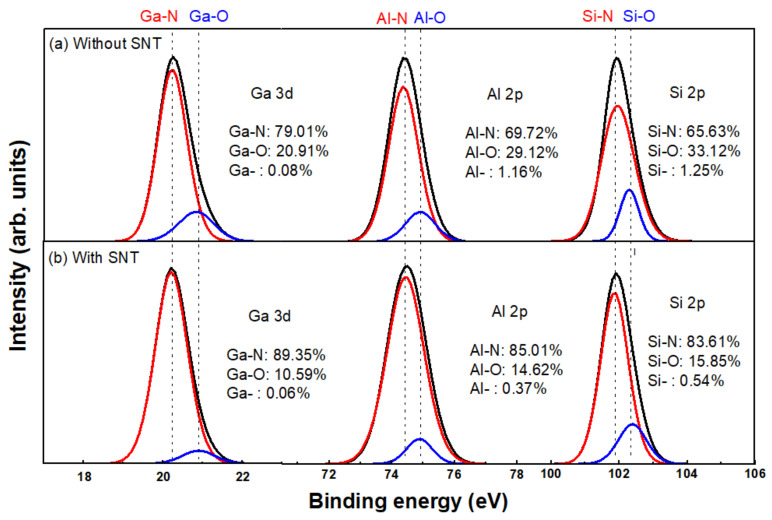
Ga 3d (left), Al 2p (middle) and Si 2p (right) core-level spectra at the Si${}_{3}$N${}_{4}$/AlGaN interface in the 8-nm Si${}_{3}$N${}_{4}$/AlGaN/GaN samples (**a**) without SNT and (**b**) with SNT. Each measured spectrum (symbol) is resolved into two Gaussian functions that correspond to M in M-N (solid red line) and M-O (solid blue line) bonds. M represents Ga (**left**), Al (**middle**) or Si (**right**). The solid blue red line is a superposition of the two fitting functions.

Figure 7 shows the Ga 3d (left), Al 2p (middle) and Si 2p (right) core-level spectra at the Si${}_{3}$N${}_{4}$/AlGaN interface in the 8-nm Si${}_{3}$N${}_{4}$/AlGaN/GaN samples, which is simulated by Avantage. For the control sample without SNT, the native oxide exhibits a large shoulder (Ga-O bonds) on the high binding energy side of the Ga-N peak [Figure 7a left]. The Ga 3d core-level spectrum in Figure 7a indicates the existence of amorphous native oxide at the Si${}_{3}$N${}_{4}$/AlGaN interface without SNT. Ga-O/Al-O/Si-O bonds may also be a kind of the interface state [19], which could lead to the V${}_{th}$ shift phenomena in GaN MIS-HEMTs. During the formation of Ga-O/Al-O/Si-O bonds, some of them are natural oxidation and some are O filled with N vacancy. With SNT, a higher intensity of Al-N bonds is observed at the Si${}_{3}$N${}_{4}$/AlGaN interface [Figure 7b middle]. The proportion of Ga-N bonds increases from 79.01–89.35%. The proportion of Al-N bonds increases from 69.72–85.01%. The proportion of Si-N bonds increases from 65.63–83.61%. At the same time, the proportion of Ga dangling bonds decreases from 0.08–0.06%. The proportion of Al dangling bonds decreases from 1.16–0.37%. The proportion of Si dangling bonds decreases from 1.25–0.54%. The change of the proportion of chemical bonds indicates that part of the N vacancies is filled, and some O atoms in Ga-O bonds are replaced by N during SNT treatment.

CO${}_{2}$ is a double bond structure with large structure with large activation energy and stable chemical properties. It does not participate in the reaction during supercritical nitridation treatment. It acts only as a solvent for supercritical NH${}_{3}$, avoiding the supercritical NH${}_{3}$ reaction and eroding the device electrodes. As mentioned above, due to the dry etch process and high growth temperature in LPCVD, the very surface of the AlGaN layer and the interface between Si${}_{3}$N${}_{4}$ and AlGaN layer may be relatively defective, which could lead to the trap-induced V${}_{th}$ shift phenomena in GaN MIS-HEMTs [20].

**Figure 8:**
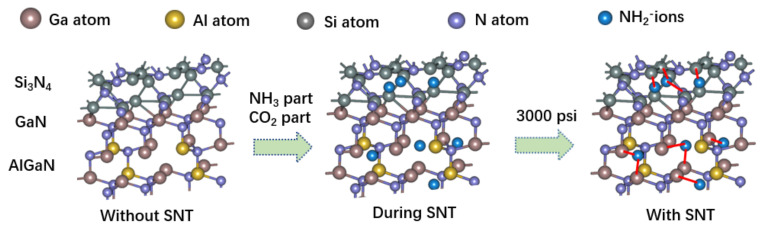
The supercritical nitridation technology model of the fabricated all-GaN integrated MIS-HEMTs.

Figure 8 shows the supercritical nitridation technology model of the fabricated all-GaN integrated MIS-HEMTs, which is simulated by Nanodcal.In the process of SNT, NH${}_{2}^{-}$ produced by auto coupling ionization of NH${}_{3}$ has strong nucleophilic ability and can fill the nitrogen vacancy near the Si${}_{3}$N${}_{4}$/AlGaN interface caused by the dry etch and high temperature process. During the nucleophilic reaction [21], NH${}_{2}^{-}$ can react with Ga-O/Al-O/Si-O to replace O and from the Ga-N/Al-N/Si-N bonds. The exact passivation mechanism during SNT is still under investigation.

## 4. Conclusions

In summary, high performance AlGaN/GaN MIS-HEMTs realized via supercritical nitridation technology has been designed, fabricated, and measured. By comparing the devices without and with SNT, we find that supercritical nitridation technology can effectively repair the defects and suppress the shift of threshold voltage. With SNT, the optimized MIS-HEMTs demonstrate a low V${}_{th}$ shift decrease of about 0.54 V at V${}_{G_{s}weep}$ from 0 V to 15 V. Our demostration of the supercritical nitridation technology to repair defects of wide-bandgap family of semiconductors may bring about great changes in the field of device fabrication.

## Data Availability

All data, models, and code generated or used during the study appear in the submitted article.
